# Characteristics, Cause, and Severity Analysis for Hazmat Transportation Risk Management

**DOI:** 10.3390/ijerph17082793

**Published:** 2020-04-17

**Authors:** Li Zhou, Chun Guo, Yunxiao Cui, Jianjun Wu, Ying Lv, Zhiping Du

**Affiliations:** 1 School of Information, Beijing Wuzi University, Beijing 101149, China; Zhoulibit@126.com; 2 Key Laboratory of Transport Industry of Big Data Application Technologies for Comprehensive Transport, Ministry of Transport, Institute of Transportation System Science and Engineering, School of Traffic and Transportation, Beijing Jiaotong University, Beijing 100044, China; 19120811@bjtu.edu.cn (C.G.); 18120782@bjtu.edu.cn (Y.C.); 3 State Key Laboratory of Rail Traffic Control and Safety, Beijing Jiaotong University, Beijing 100044, China; jjwu1@bjtu.edu.cn; 4 School of Logistics, Beijing Wuzi University, Beijing 101149, China; du_zhiping@126.com

**Keywords:** Hazmat transportation, accident characteristic, decision tree, F-N curve, uncertainty

## Abstract

The accidents caused by hazardous material during road transportation may result in catastrophic losses of lives and economics, as well as damages to the environment. Regarding the deficiencies in the information systems of hazmat transportation accidents, this study conducts a survey of 371 accidents with consequence Levels II to V involving road transportation in China from 2004–2018. The study proposes a comprehensive analysis framework for understanding the overall status associated with key factors of hazmat transportation in terms of characteristics, cause, and severity. By incorporating the adaptive data analysis techniques and tackling uncertainty, the preventative measures can be carried out for supporting safety management in hazmat transportation. Thus, this study firstly analyzed spatial–temporal trends to understand the major characteristics of hazmat transportation accidents. Secondly, it presented a quantitative description of the relation among the hazmat properties, accident characteristics, and the consequences of the accidents using the decision tree approach. Thirdly, an enhanced F-N curve-based analysis method that can describe the relationship between cumulative probability *F* and number of deaths *N*, was proposed under the power-law distribution and applied to several practical data sets for severity analysis. It can evaluate accident severity of hazmat material by road transportation while taking into account uncertainty in terms of data sources. Through the introduction of the as low as reasonably practicable (ALARP) principle for determining acceptable and tolerable levels, it is indicated that the F-N curves are above the tolerable line for most hazmat accident scenarios. The findings can provide an empirically supported theoretical basis for the decision-makers to take action to reduce accident frequencies and risks for effective hazmat transportation management.

## 1. Introduction

Hazardous materials (hazmats) not only play crucial roles in critical areas of industrial sectors, such as heavy metal industries, agriculture, pharmaceutical industries, and oil and fuel industries, they also constitute a significant part of our domestic lives [1,2,3,4]. An unprecedented boom in hazmat usage has resulted in a dramatic growth in accidents caused by road hazmat transportation. The degree of danger of transport together with the risk of life is increasing. In light of possible catastrophic losses of lives and economics, as well as damages to the environment, there is an urgent theoretical and practical need to reduce hazmat transportation accidents (HTAs) associated with consequence and severity, which has drawn increasing concerns from the public [5,6,7]. In contrast to general traffic accidents, HTAs can lead to more severe results due to the inherent physical and chemical instabilities of the substances during the road transportation process. Thus, the catastrophic accidents involving road hazmat transportation have often been termed as low probability with high consequence. Previous studies have examined the social risk generated from hazmat transportation. However, more research is needed regarding how the decision-makers should react to the implications of the severity of the accident consequences, and which factors/aspects should be paid more attention since they could intuitively contribute to the HTAs. These problems can be solved by proposing a comprehensive analysis framework with integrations of multiple effective methods to understand the accident characteristics, causes, and severities for implementing adaptive management policies. Meanwhile, concerning the inherent uncertainty existing in the database, which may lead to fails in decision making, more advanced severity analysis and evaluation methods are required to be developed to address the complexity that has been ignored in previous studies. Moreover, the influencing factors that affect accident consequences are miscellaneous, and they interact in complex ways, making it difficult to disentangle the relationships between accident consequence and the variables by conventional methods, especially in the transportation of hazmats. The application of machine learning techniques is desired and challenging in the field of HTA, which can be expected to achieve a relatively satisfying performance in cause–consequence analysis.

Moreover, China is a major country producing and using chemicals, whose outputs of several species of hazmats rank first all over the world. The hazmat industry has become one of the pillar industries of China’s national economy, whose market scale is estimated to reach 10.128 × 10^9^ Chinese Yuan in the next five years; meanwhile, the gross benefit of hazmats keeps growing at an annual average rate of around 5%. Accordingly, increasing numbers of HTAs in recent years has put the issue of hazmat transportation management high on China’s public and policy agendas. It calls for public, comprehensive, and accurate data on which an effective HTA management system can be constructed. In terms of HTAs in China, no specialized accident database is available, much less investigation that clarifies China’s current situation.

The objective of this work is to develop an integrated research framework for HTA associated with risk analysis. It firstly presents the trends and the characteristics of fatal HTAs to identify the related affecting factors between the years 2004 and 2018. Based on the analysis, the study models the accident causes and the corresponding consequences using the decision tree approach. Then, an enhanced F-N curve-based analysis method is proposed under uncertainty and is applied to several hazmat transportation-related data sets. The F-N curve is one of the important tools for severity analysis and risk judgment by describing the relationship between cumulative probability *F* and number of deaths *N*. These results are compared for comprehensively evaluating the severity and understanding of the associated implications of HTAs. 

In response to the current research challenges, the main contributions of the study include the following three aspects. First, the study proposes a comprehensive research framework for characteristics, causes, and severities analysis of HTAs, so that the adaptive management policies can be implemented. Second, the study develops an enhanced F-N curve establishment method while addressing the inherent uncertainty in severity analysis. With the introduction of the as low as reasonably practicable (ALARP) principle, the possible severity of hazmat accidents by road transportation can be evaluated under certain confidence levels, which has been ignored in previous research. Third, the study extends the machine learning techniques in the application of hazmat transportation management, with the integration of multiple data analysis methods into the proposed research framework. In particular, the decision tree method is used to quantitatively describe the complex relationship between the accident consequences and key influencing factors.

The rest of the study is organized as follows. Section 2 presents the state of the art that is divided into three subsections. Section 3 describes the source of HTA data. Section 4 summarizes the research methods. Section 5 analyzes the temporal and spatial characteristics of HTAs. Then, we further investigate the relation among the influencing factors and HTA consequences using the decision tree approach in Section 6. Section 7 presents the proposed enhanced F-N curve method under uncertainty and the applications to several practical data sets for severity analysis. Section 8 presents the discussions. Finally, we conclude this article in Section 9. 

## 2. State of the Art

In the section, we review studies that are directly related to the objectives of this paper, focusing particularly on the characteristics of HTA, cause–consequence analysis, and severity analysis. The following subsections briefly introduce each stream of research. 

### 2.1. Characteristics of HTAs

The analysis of the characteristics of HTAs is an important issue in accident prevention because it can provide fundamental information on understanding the causes and severity of accidents. There is a large amount of research using statistical methods to identify the factors that influence the HTAs such that several historical surveys of accidents have been carried out, revealing their characteristics including frequency, temporal and spatial trends, as well as consequences [8,9,10,11,12,13]. Oggero et al. [8] surveyed 1932 accidents that occurred during the transport of hazardous substances by road and rail, and demonstrated that there was an increase in the accident frequency over time. Some research has focused on HTA analysis in China. For example, a survey on hazmat accidents during road transport in China from 2000 to 2008 conducted by Yang et al. helped to understand the spatial trends of accident frequencies [9]. Shen et al. [10] specifically surveyed tank truck accidents involved in road hazmat transportation in China from 2004 to 2011, including causes, locations, types, time of occurrence, hazard classes for materials involved, consequences, and the corresponding probabilities. The studies on the topic that focus on using statistical methods to describe specific event attributes and trends of HTAs can provide a basis for further accident data mining involving hazardous materials transportation. Research such as consequence prediction and risk analysis can be carried out based on the above analysis of HTA characteristics.

### 2.2. Cause–Consequence Analysis

Many methods for analyzing the relationships among the causes that may lead to accidents and the corresponding possible consequences are available, which can be categorized as statistic regression models (i.e., multivariate regression and multinomial logit) [1,14,15,16,17,18] and machine learning methods (typical as Bayesian network and decision tree) [19,20,21,22,23]. Desai et al. [1] presented a sequence of statistical models (i.e., partial least squares, spline regression, and Box-Cox transformations) to estimate the population affected and the impact of the cost incurred as a result of hazmat accidents. Zhao et al. [20] studied the factors that influence HTAs through Bayesian networks based on expert knowledge using Dempster–Shafer evidence theory to help reduce accident risks. Amirfarrokh et al. [22] made a comparison of four statistical and machine learning methods for crash severity prediction. As for these methods, the statistical methods always require a predefined mathematical expression between dependent and independent variables, which can be difficult when dealing with complex relations with combinations of linear and non-linear features. The Bayesian network approaches, generally have higher data qualities and computational complexities. The decision tree learning technique is a supervised data mining method, that can serve as an effective tool for multivariate data analysis. It thus can be used to explore the relationships of a large number of candidate input attributes to an output (target) attribute, making it suitable for conducting complex relation analyses among the causes and the consequence [23]. Thus, the decision tree methods have been reported in the road safety field and found a significant association for crashes and factors such as geometric patterns and factors, crash seriousness, and over speeding [23,24,25,26,27,28,29]. Specifically, Abellán et al. [24] applied the decision tree method to extract the decision rules for traffic accident consequence analysis. Shah et al. [25] proposed a two-stage decision making approach for analyzing road safety performance, wherein the decision tree was used to identify the contributing factors. However, unlike the ordinary road traffic accident data, the database of HTAs usually contains a large number of records with incomplete information and multiple features from the hazmats. Besides the factors in terms of drivers, vehicles, roads, and environment, the transported hazmats can also affect the accident consequences; these may result in highly complexities in modeling. Hence it is challenging to apply such a method to extend the existing accident analysis. The literature on the key influencing factors in relation to the consequences of accidents for hazmat road transportation appears to be limited.

### 2.3. Severity Analysis

To bring HTA risks under effective control, comprehensive, standardized, and georeferenced information on hazmat risks and accidents is crucial for decision making, supervision, assessments, insurances, and management. In addition to analyzing HTA characteristics associated with accident causes, research also needs to focus on accident severity. The HTA severity can be measured by different methods, such as casualties, property damage, or environmental impact. However, there are many situations in which it is difficult to employ cost–benefit analysis because it is not possible to either identify in advance or to value all the major consequences of accidents. For example, HTAs may have repercussions that go much wider than their direct effects on people and property. Thus, the F-N curve (i.e., a curve relating the probability *F* per year, causing *N* or more fatalities) as a workable societal risk criterion is adapted for HTA severity analysis. The F-N curve is one of the important tools for severity analysis and risk judgment by presenting the relationship between the accumulated probability and the number of deaths [30,31,32,33]. For example, Caliendo and De Guglielmo [34] analyzed the risk regarding dangerous goods vehicles running through road tunnels. They found that the social risk in terms of the F-N curve showed a raised risk level with an increased peak hour traffic volume, the percentage of heavy goods vehicles, and a failure of the emergency ventilation system. Pompone and de Oliveira Neto [35] analyzed the societal risk of accidents of road transportation of hazmats from 1983 to 2015 in Brazil using F-N curves. Furthermore, to explore the acceptable risk standard value of social risk, the ALARP principle with the framework of acceptable, tolerable, and unacceptable risk intervals was used. It is usually used in the existing research, and was first proposed by the UK health and safety commission. Along with the ALARP principle, the F-N curve method enables us to understand the societal risk level and, where appropriate, allows comparison with the relevant tolerability risks under different scenarios. For example, Fabiano et al. [36] presented a site-oriented framework for risk assessment, wherein the acceptability risk level was set down by F-N curves established in the Netherlands. Zhang et al. [37] established F-N curves of gas accidents and risk tolerability criteria based on ALARP. It was indicated that tolerability criteria in developed countries are stricter than those in China. Vidmar and Perkovič [38] studied the evolution of risk acceptance and evaluated risks through F-N curves of different tanker sizes for the safety assessment of crude oil tankers. However, currently published studies are yet to take into account the uncertainty while establishing F-N curves. The existing uncertainty may influence the F-N curve formulation associated with a social risk evaluation. Thus, it is important to narrow this knowledge gap by proposing an enhanced F-N curve that goes beyond traditional deterministic approaches for analyzing the severity of the HTAs.

## 3. Data Collection

To illustrate and assess the usefulness and limitations of the existing datasets for HTA analysis, we collected and analyzed data and information on the accidents by road transportation with Levels II to V over the past 15 years (2004–2018). Generally, accidents can be divided into five levels, which are Levels I to V based on the casualties or/and economic losses according to the Byelaw Governing Reporting, Investigation, and Handling of Production Safety Accidents of China. The classified five levels as follows: (1) Level I means no deaths and serious injuries; (2) Level II refers to an accident that leads to less than 3 deaths, or less than 10 serious injuries, or direct economic losses of less than 10 million yuan; (3) Level III means an accident that causes the death of more than 3 people, but less than 10 people, or the serious injury of more than 10 people but less than 50 people, or the direct economic losses of more than 10 million yuan but less than 50 million yuan; (4) Level IV means an accident that causes more than 10 but less than 30 deaths, or more than 50 but less than 100 serious injuries, or direct economic losses of more than 50 million but less than 100 million yuan; (5) Level V means an accident that causes more than 30 deaths, or more than 100 serious injuries, or a direct economic loss of more than 100 million yuan. Among them, Level I is the lowest level without casualties, and Level V is the severest one with serious casualties or/and economic losses. However, as of now, there is no public and free database of HTAs that can offer complete information covering the required spatial, temporal, and detailed contexts of accident descriptions, making it difficult to obtain relevant statistical data. In the study, we review and extract the useful information regarding HTAs by road mainly from the following resources, specifically several official websites (e.g., the Ministry of Transport, the Ministry of Emergency Management, and the Ministry of Environmental Protection of China), useful data and information collected from professorial websites (e.g., the National Registration Center for Chemicals, the China Chemical Safety Association of China and State Administration of Work Safety, and safety management), reports from journal publications (e.g., Chemical Safety and Environment), and other online news. Moreover, since some accident records are brief and incomplete, we also additionally searched the internet for complementary information item by item.

**Figure 1:**
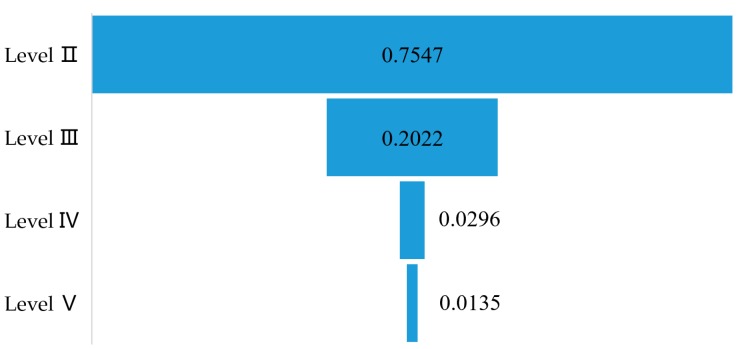
The occurrence probabilities of 371 hazmat transportation accidents (HTAs), with levels classified according to the casualties or economic losses caused by accidents.

Accordingly, HTAs were identified from more than 13,000 road chemical accidents from the above of mentioned sources. Only accidents of Levels II to V, for which the number of deaths is known, were included in the analysis. Finally, a total of 371 HTAs were singled out for review, all of which occurred during transportation, excluding the accidents that occurred during loading, unloading, and maintenance. Information on each HTA record is given concerning the hazmat classes and species of hazmat, United Nations (UN) number, the locations, time, date, types, impacts (leaking, non-leaking, fire, explosive, poisonous, corrosive, and others), and consequences (e.g., levels, casualties, and the direct economic losses to property), as well as specific detailed descriptions of the accident. In detail, the accident occurrence probabilities for Levels II to V are shown in Figure 1. It is shown that the accident level is negatively related to its proportion, the higher the accident level, the lower the occurrence. The proportion of accidents at Level II was the highest, accounting for 75.47% of HTAs. Particularly accidents belonging to Levels IV and V account for less than 5% in total.

## 4. Methods

**Figure 2:**
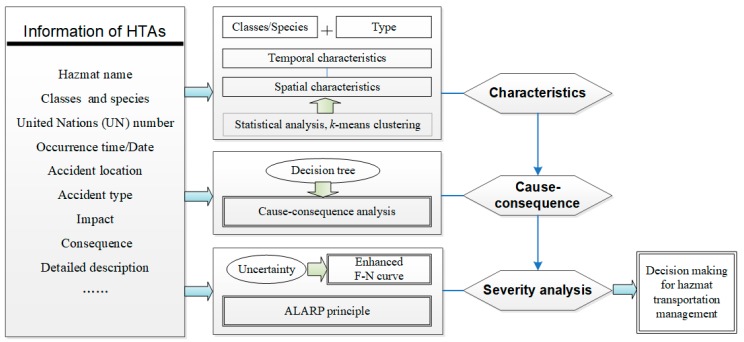
Study framework layout. In the figure, the F-N curve describes the relationship between the cumulative probability *F* and the number of deaths *N*. The ALARP principle is the abbreviation of as low as the reasonably practicable principle.

The study introduces multiple analytical methods to support the development of a comprehensive research framework for the accident characteristics, causes, and severities analysis, so that adaptive management policies can be implemented. The method used in the study includes the statistical analysis, the *k*-means clustering, the decision tree model, and the F-N curve method associated with the ALARP principle. The proposed analytical framework with the integration of research methods is summarized in Figure 2.

In detail, in the characteristics of the HTAs section, the data analysis method based on the statistics and probability theory was applied to explore the distribution of hazmat classes, causes, and frequencies of HTAs, which has been widely used in the existing literature [8,9]. The *k*-means clustering method, serving as a prototype of the cluster that can partition the input data set into *k* partitions (clusters), was introduced for spatial characteristics analysis. It addresses the multiple socio-economic factors that influence the number of accidents for each province/city, and thus, can help the decision-makers understand the importance of managing serious accidents based on the spatial characteristics of HTAs. Then, the decision tree model, as a suitable machine learning method, was used for illustrating the complex relationship existing in the cause–consequence analysis compared with conventional methods (i.e., statistic regression models and Bayesian network). Although it has been reported in the road safety field, the applications in examining the key influencing factors in relation to the consequences for hazmat road transportation accidents is still somewhat limited [28,29]. The study is an extension of the existing accident analysis. In the severity analysis section, an enhanced F-N curve was adapted under uncertainty by presenting the relationship between the accumulated probability and the number of deaths. As for the other severity measurement indicators, such as casualties, property damage, or environmental impact, it may be difficult to employ cost–benefit analysis because it is not possible either to identify in advance or to value all the major consequences of accidents. Moreover, the existing applications of F-N curves rarely consider the inherent uncertainty in the database, which may influence the F-N curve formulation associated with a social risk evaluation and lead to failures during decision making. The ALARP principle allowed us to evaluate the relevant tolerability risks under different scenarios and make the result of severity evaluation practical significance [36,38].

## 5. Analysis of HTAs Associated with Temporal and Spatial Characteristics

In the section, the 371 HTAs of Levels II to V obtained in the previous section were used for conducting the accident frequency, as well as the temporal and spatial characteristics analysis.

### 5.1. Accident Frequency Characteristics

**Figure 3:**
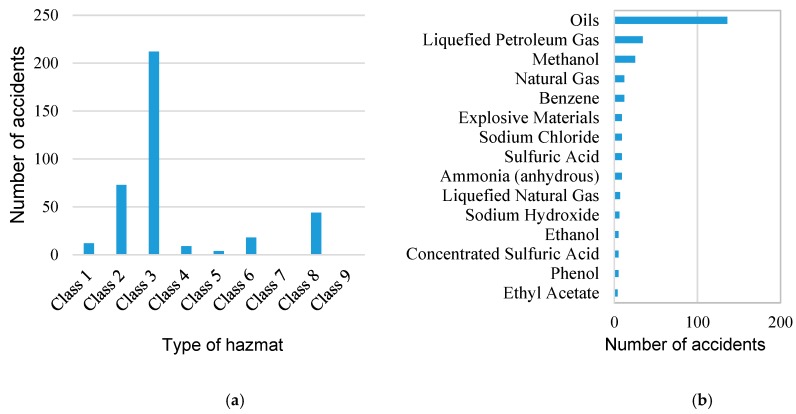
Hazmat classes and species statistics of the 371 HTAs. (**a**) The number of accidents for each class; and (**b**) the top 15 of hazmats involved in the 371 HTAs.

According to the European Agreement Concerning the International Carriage of Dangerous Goods by Road, hazmats can be divided to nine categories, which are: Class 1, explosive substance and articles; Class 2, gases; Class 3, flammable liquids; Class 4, flammable solids, self-reactive substances, substances liable to spontaneous combustion, and substances which in contact with water emit flammable gases; Class 5, oxidizing substances and organic peroxides; Class 6, toxic substances and infectious substances; Class 7, radioactive materials; Class 8, corrosive substances; and Class 9, miscellaneous hazardous substances [39]. As for the statistical accidents obtained as mentioned in the previous section, radioactive substances and miscellaneous hazardous substances are not involved. The class and species statistics of the HTAs are shown in Figure 3. Figure 3a shows the number of accidents for each hazmat class, wherein the classes 7 and 9 are not covered in the investigated accidents. Figure 3b shows the top 15 hazmat species and the corresponding number of accidents. Due to a tremendous amount of freight volume and the properties of Class 3 hazmats transported in the tank, there were more Class 3 HTAs than other classes of hazmats. Oil, as a typical flammable liquid of Class 3, was one of the most common hazmats by road transportation. As can be seen from Figure 3b that oil-related HTAs ranked at the top of the figure. The flammable gases of Class 2 (e.g., liquefied petroleum gas and natural gas) and some other flammable liquids of Class 3 (e.g., methanol and benzene) also accounted for a large proportion of the investigated HTAs.

**Figure 4:**
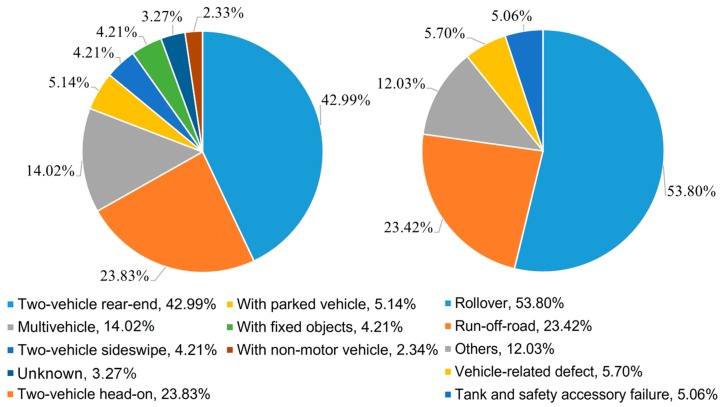
Collision and non-collision of the 371 HTAs.

HTAs can be divided into collision and non-collision accidents. According to the data statistics, the collision and non-collision accidents accounted for 57.68% and 42.59%, respectively (shown in Figure 4). It is indicated that even if without vehicle collision, there could be more than a 40% possibility of causing lethal HTAs. In detail, the major types of collision accidents were two-vehicle rear-end and two-vehicle head-on accidents, accounting for 42.99% and 23.83% of collision accidents, respectively. The head-on collisions were more likely to occur in places with complex road conditions or poor terrains, such as intersections or steep slopes of curves. In contrast, the major types of non-collision accidents were rollover and run-off-road, accounting for 53.80% and 23.42% of non-collision accidents, respectively. Among them, rollover accidents were prone to occur on steep slopes, bridge culverts, and other poor road conditions. The accidents were often accompanied by leaking combustion. Overall, the accident type with the most probability was two-vehicle rear-end, accounting for 24.80%, followed by rollover (22.91%). As transported hazmats are generally of large mass and high inertia, trucks could be prone to rear-end collisions or rollover accidents when approaching a curve.

### 5.2. Temporal Characteristics of HTAs

**Figure 5:**
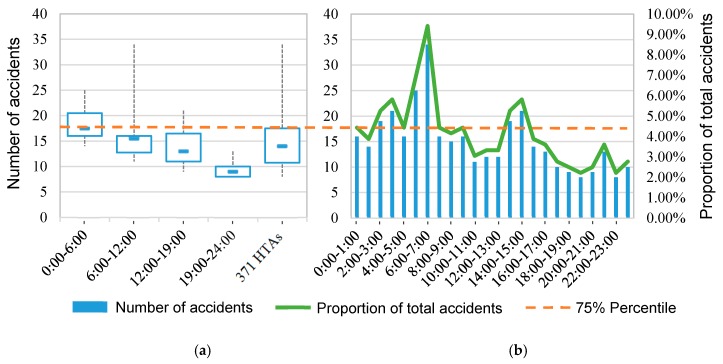
Temporal distribution of the 371 HTAs. The number of accidents is counted according to the time period. (**a**) describes the upper quartile, the lower quartile, and the median of the accident number for the four periods and the full sample. In Figure (**b**), the bars show the number of accidents in each hour, and the green line presents the corresponding proportion of the total 371 HTAs. The dotted line is the 75% quartile of accidents for the full sample, with a value of 17.5.

As for the temporal characteristics, the daily and monthly variations of HTAs were investigated in the study. The daily HTA trend is shown in Figure 5. It is indicated that the number of HTAs in the early morning was relatively high, with the significant periods during 2:00–4:00 am and 5:00–7:00 am. The accident number in the afternoon varied greatly, however the period from 13:00–15:00 also showed a higher frequency of HTAs, exceeding 17.5 which was the 75% quartile of the accident samples (Figure 5a). It is mainly due to fatigue driving, as most drivers are likely to be exhausted during these periods.

**Figure 6:**
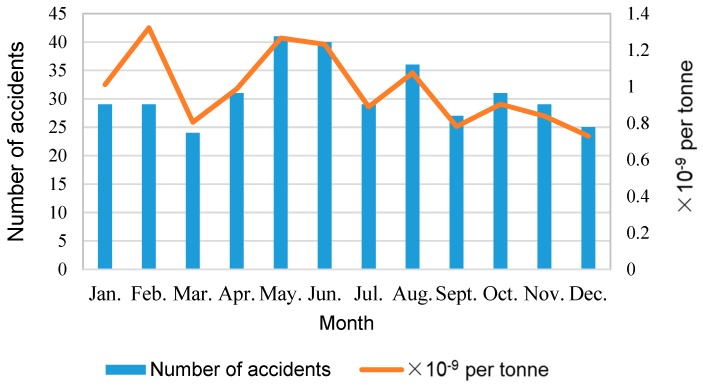
Monthly distribution of HTAs.

Figure 6 shows the monthly characteristics of HTAs. In the figure, besides the total accident amount for each month, we also calculated the accident rate per unit freight volume. The value can be obtained by dividing the total number of accidents in a month by the total volume of transportation in the corresponding month, shown as the orange curve in Figure 6. In Figure 6, more HTAs appeared in May, June, and August, and correspondingly, these months are also the peak periods for hazmat transportation, with relatively larger transportation volumes. In these months, the inherent instability of hazmats, along with the rise of the environmental temperature, may increase the risks of accidents. Notably, the accident rate per unit freight volume in February was the highest. The freight volume in the month was relatively low due to the Chinese New Year. Thus, although the total number of accidents was not large, the value obtained through dividing the accident number by the transportation volume could be the highest. Overall, it requires enough attention to be paid by the decision-makers.

### 5.3. Spatial Characteristics of HTAs

**Figure 7:**
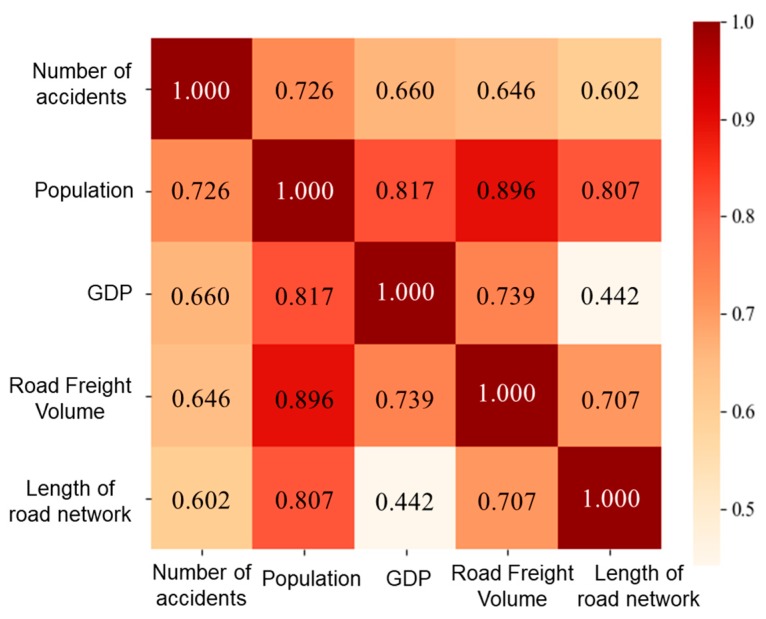
The correlations between population, GDP (gross domestic product), road freight volume, length of road network, and the number of accidents.

In order to study the spatial characteristics of HTAs in different provinces, the related social and economic data of 31 provinces and cities from 2004–2018 were also investigated. In the study, we considered the HTAs by road in the Chinese mainland and did not include those that occurred in Hong Kong, Macao, or Taiwan. Considering the possible influence on the accidents by the spatial factors, social and economic data were collected for these provinces and cities. The correlation between the HTA and the social and economic indicators were examined. The indicators of the regional population, gross domestic product (GDP), road freight volume, and length of road network were correlated to the number of accidents, with the correlation coefficients higher than 0.6 (Figure 7). Meanwhile, the correlations among these indicators were also calculated. As shown in Figure 7, among the indicators, GDP and length of road network are weakly related to each other and were selected as cluster features for analyzing the spatial distributions of HTAs. 

**Figure 8:**
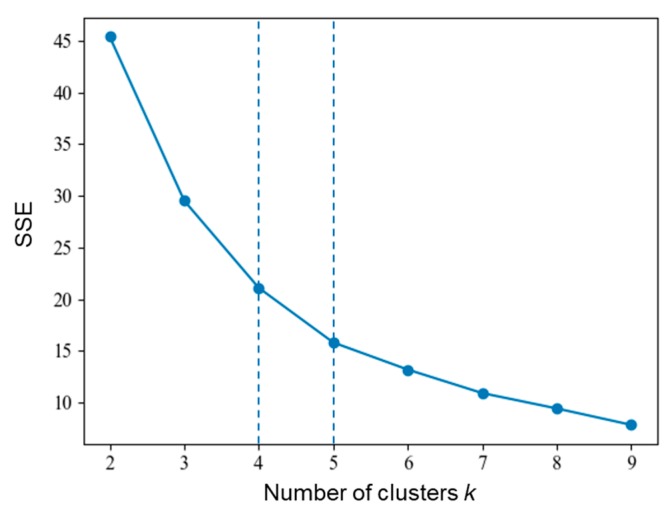
*k* selection using the Elbow method, suggesting *k* equal to 4 or 5.

To conduct the *k*-means cluster analysis, the Elbow method was used for identifying the cluster number of *k* [40,41]. The idea of the Elbow method is to run *k*-means clustering on the dataset for a range of values of *k*, and for each value of *k* calculate the sum of squared errors (SSE). Then, a line chart of SSE for each value of *k* can be obtained (shown in Figure 8). The location of a bend (knee) in the plot is generally considered as the indicator of the number of the appropriate clusters. It is noted that the Elbow method is sometimes ambiguous, and thus, multiple candidates of *k* can be considered. In Figure 8, the distortion begins to go down slowly around *k* = 4 and 5; hence the location of a bend can be suggested as 4 or 5. Thus, the number of clusters needed for the data set was selected as 4 or 5. 

Subsequently, the clustering results with *k* = 4 and 5 were both tested by analysis of variance (ANOVA) and Tukey’s test so that the reasonable number of *k* could be obtained. The study set a significance level of 0.05. In detail, ANOVA was used to compare the mean values of two or more groups of samples for significant differences. The *p*-values of *k* = 4 and 5 obtained from the ANOVA indicated that the Tukey’s test was available for proceeding to multiple comparisons. Tukey’s test is a method of multiple (≥ 3) comparisons between groups and compares differences between each pair of means; this can help detect whether there are statistically significant differences among cluster averages. Like both the *t*-test and ANOVA, Tukey’s test assumes that the data from the different groups comes from populations where the observations have a normal distribution and the standard deviation is the same for each group [42]. Table 1 shows the test results of clustering when *k* = 4 and 5, wherein the average comprehensive factor score was used to present the performance of each case. When *k* = 5, the five clusters were divided into four groups. Although there are significant differences between pairs of Clusters 1, 2, and 5, no significant difference exists between Clusters 3 and 4 as the *p*-value of 0.143 exceeded 0.05. In comparison, the clustering result of *k* = 4 was more appropriate, as the difference between each pair of clusters was significant and there was no pair of clusters that were grouped together. 

**Figure 9:**
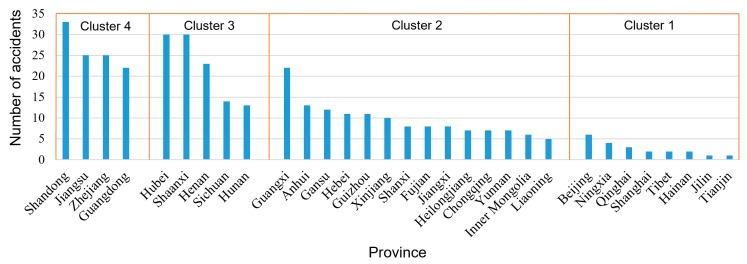
Clustering result dividing 31 provinces/cities into four groups.

A total of four clusters of provinces and cities were grouped by clustering, as shown in Figure 9. There were significantly more accidents in Shandong, Hubei, Shaanxi, Jiangsu, Zhejiang, Guangdong, Henan, and Guangxi than the other provinces, with more than 20 accidents in each of these provinces. Moreover, the four groups divided through the clustering method, as shown in Figure 9, were not entirely coincident with the ranking result based on the number of accidents. As the selected criteria were positively correlated with the HTAs, the clustering result of four groups could help identify the importance of controlling accidents by not relying solely on the numbers of the accidents. For example, although Guangdong ranked 7th in the number of accidents, the clustering result shows that the province was divided into Cluster 4, representing the most important group in controlling severe accidents because of its higher GDP. In contrast, Hubei and Shaanxi ranked 2nd and 3rd based on the statistic accidents of Levels II to V, but they are grouped in Cluster 3. Moreover, Liaoning is divided into Cluster 2, while the number of accidents in Liaoning was lower than that in Beijing (which held the top in Cluster 1). With full consideration of the aspects of GDP and length of road network, Beijing, despite higher in the number of accidents, was clustered into the last group.

**Figure 10:**
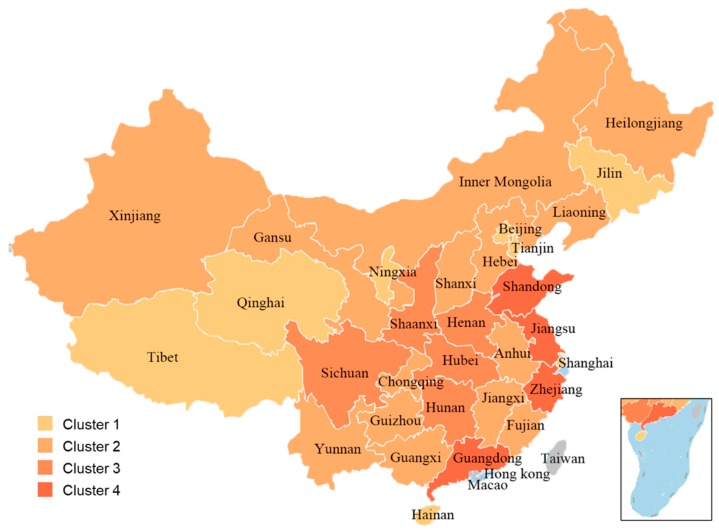
The geographic clustering results.

From a regional perspective, the areas with more severe accidents were generally the coastal areas of eastern China, southern China, and central China, with more details geographically displayed in Figure 10. Specifically, the accidents in eastern China accounted for the most significant proportion (30.73%), followed by central China (17.79%) and northwest China (15.90%).

## 6. Decision Tree-Based HTAs Cause–Consequence Analysis

In order to understand the correlation among the hazmat properties, accident characteristics, and the consequences of HTAs, the decision tree method was used to identify which characteristics define the specified groups, and to outline which specific variables have the most substantial impact on group differentiation. A decision tree is a tree-like collection of nodes intended to create a decision on values affiliation to a class or an estimate of a numerical target value. Each node represents a splitting rule for one specific attribute. For classification, this rule separates values belonging to different classes. Generally, the chi-squared automatic interaction detection (CHAID) analysis is one way to determine how variables combine to determine group membership [23]. It is based on the target variable (independent variable; the independent variables of CHAID can only be classified) self-hierarchical tree structure. The root node is the dependent variable. The predictors continuously generate parent and child nodes based on the degree of chi-square significance. The higher the chi-square significance, the more variable the predictor becomes the root node. The CHAID operator provides a pruned decision tree that uses a chi-squared-based criterion instead of information gain or ratio criteria gain, which is useful when the various existing predictive variables are classification ones. In the study, the consequence level of accidents was the dependent variable, and hazmat class, the accident type, the time and the cause of accidents, and road level were independent variables. The variables, with the exception the time, were all specified as classification variables. As some accidents may contain more than one class of hazmat, the study separated different classes into multiple accident cases. For example, if an accident contained two classes of hazmats and resulted in an accident consequence of Level II, it was separated into two accidents of Level II with different inputs of hazmat classes. Thus, the accident inputs at the root of the decision tree were more significant than the actual number of HTAs. The detailed classification of independent variables prepared for modeling the decision tree is presented in Table 2. The last column of the table provides the input number of accident cases; in particular, the HTAs containing two or more hazmat classes have been separated into multiple cases. 

**Figure 11:**
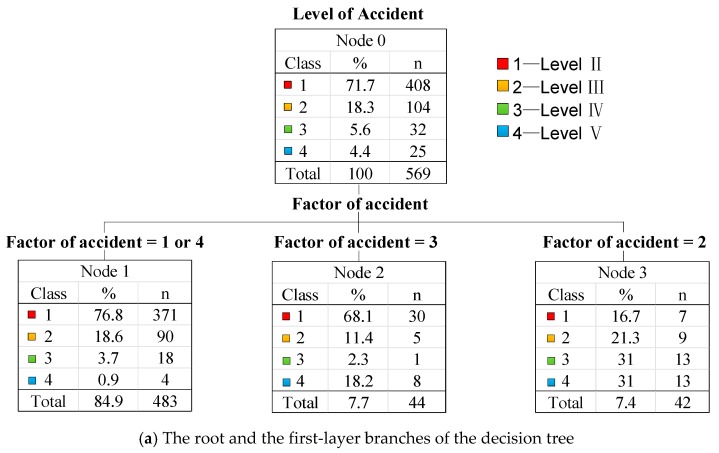

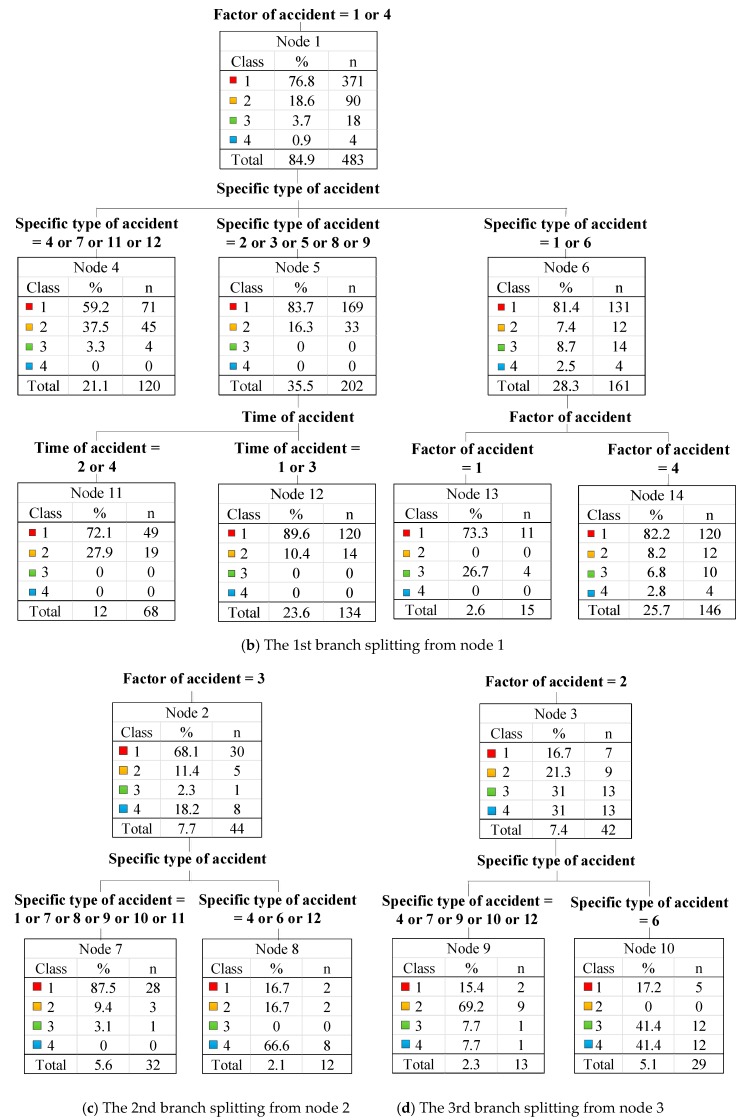
The decision tree model with 14 nodes, wherein Class 1, 2, 3, and 4 in each node corresponds to Level II, Level Ⅲ, Level Ⅳ, and Level V of HTAs.

To interpret the complex relationship among multiple variables, the decision tree model was formulated consisting of four subgraphs, as shown in Figure 11. Considering the accident separation, a total of 569 accident cases could be input at node 1. Among them, Figure 11a represents the root and the first-layer branches of the decision tree. It is shown that the root node is split into three branches based on the causes of accidents in the study. Then, the first-layer branches perform as new root nodes which leads to more branches for further elaboration of the relations between the causes and the consequences of the accidents. Figure 11b–d are respectively the 1st, 2nd, and 3rd branches generated from the root of the decision tree.

Taking node 2 generated from the root shown in Figure 11a as an example, node 2 represents the accidents caused by equipment malfunction, with a total of 44. The decision tree then classified the specific types of accidents 1, 7, 8, 9, 10, and 11 to node 7 in Figure 11c, which represents the number of accidents caused by factor 3, that is, equipment malfunction As shown in Figure 11c, based on the data of HTAs that belong to the specific types and the corresponding proportion of different consequence levels, there was a 5.6% chance that can lead to the specific types of accidents at node 7, that is, collision with fixed objects, multivehicle collide, rollover, run-off-road, tank and safety accessory failure and vehicle-related defect. If it happens, there was an 87.5%, 9.4%, and 3.1% chance for Level II, III, and IV accidents occurring, respectively. Figure 11c also displays that the specific types of accidents 4, 6, and 12, that is, two-vehicle head-on, two-vehicle rear-end and other specific types, were classified to node 8, representing a chance of 2.1% that can result in the specific type of accident at the node. If it happens, the probabilities were 16.7%, 16.7%, and 66.6% for Levels II, III, and IV of accidents, respectively.

Moreover, a confusion matrix (Table 3) was used to visualize the performance of cause–consequence analysis by using the decision tree, which displays whether the system is confusing two classes (i.e., commonly mislabeling one as another). For example, there were 399 correct predictions in the first line; meanwhile, nine cases were incorrectly predicted to other levels. Thus, the correct prediction percentage was 97.8%. Table 3 shows that the overall accuracy evaluation of the results was 75.2%, indicating that the decision tree model resulted in a relatively satisfying interpretation of the relationship among the hazmat properties, accident characteristics, and the consequences of HTAs.

## 7. HTA Severity Analysis Using an Enhanced F-N Curve Under Uncertainty

For identify the severity of HTAs, an F-N curve was used to describe the relationship between cumulative probability and the number of deaths, which can be provided as follows [32]:

(1) $$P_{f}\left(x\right)=1-F_{N}\left(x\right)=P\left(N>x\right)=\int_{x}^{\infty }f_{N}\left(x\right)dx,$$

where $P_{f}\left(x\right)$ is the probability of more than *x* fatalities; $f_{N}\left(x\right)$ is the probability density function of the number of fatalities; and $F_{N}\left(x\right)$ is the probability distribution function of the number of fatalities, signifying the probability of less than or equal to *x* fatalities. 

In real-world practice, the probability of accidents is usually replaced by the frequency, and the accidents are grouped according to the number of deaths. Thus, the F-N curve can be presented as:

(2) $$F=1-F_{N}\left(x\right)=\frac{\sum_{i=j}^{n}N_{i}}{\sum_{i=1}^{n}N_{i}},$$

where *N* is the number of fatalities, *n* is the total number of groups, and *N_i_* is the number of accidents in group *i*, and *F* is the cumulative frequency of *N* or more fatalities.

Let *C* be the constant that determines the limit line, and *a* be the steepness of the limit line. Thus, the F-N criterion curve can be presented as follows:

(3a) $$F=\frac{C}{N^{a}}.$$

On a log-log basis, the equation is derived as follows:

(3b) lg F=−alg N+lg C.

Generally, the F-N curve is displayed as a straight line with a precise slope of −*a*. The slope values are different in various accident conditions, and the number of deaths ranges in the broader interval; using a precise slope may underestimate or overestimate the social risks of accidents. To overcome the problems, the F-N curve under uncertainty can be proposed with the slope of −*a* obeying a certain type of distribution. It will help the authorities to evaluate the risks under different accident levels comprehensively. Therefore, to better present this distribution in quantification and visualization, a method for specifying the F-N curve under uncertainty was developed for the best-fitted curve selection using the dataset of HTAs, which was mainly under the considerations of the normal, improved normal, and power-law distributions. Table 4 presents the data for F-N curves fitting under uncertainty. More details are presented as follows.

(a) The probability density function for the normal distribution is present as follows:
  (4a) $$f\left(x\right)=\frac{1}{\sigma^{\sqrt{2\pi \;}}}exp\left[-\frac{{\left(x-\mu \right)}^{2}}{2\sigma^{2}}\right],$$
  where µ is mean value and σ is the standard deviation.

**Figure 12:**
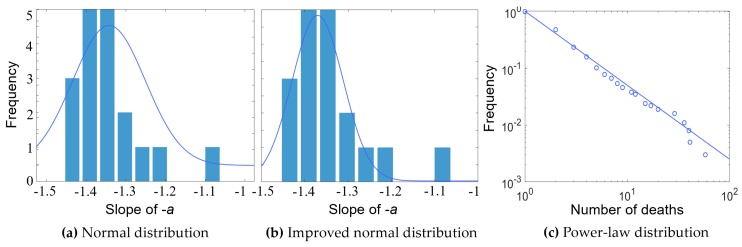
F-N curves (i.e., the relationship between the cumulative probability *F* and the number of deaths *N*) fitting based on different distributions. (**a**,**b**) demonstrate the frequency distribution histogram of slope −*a* fitting under the normal distribution and the improved normal distribution, respectively. (**c**) is the F-N curve of deaths and cumulative probability under the power-law distribution in the logarithmic coordinate system.

On a log–log basis, the cumulative probability $F_{j}$ and $N_{i}$ of the death toll in HTAs associated with the logarithmic values are calculated to specify the distribution of the slope with the corresponding mean value µ and standard deviation σ. Figure 12a shows the fitting of the normal distribution according to the database. From the confidence interval and the Equation (4a), the ratio of 10 or more accidental deaths and 100 or more accidental deaths ranged from 14.56–33.25.

(b) The improved normal distribution. Concerning the traditional deficiencies, an improved normal distribution is proposed as follows:
  (4b) $$f\left(x\right)=B+\frac{D}{{}^{\sigma \sqrt{\frac{\pi }{2}\;}}}exp\left[-\frac{2{{\left(x-\mu \right)}^{2}}^{}}{\sigma^{2}}\right],$$
  where *B* and *D* are adjusting parameters that can be obtained through the fitting process.

Similarly, the mean value µ and the standard deviation σ, as well as the distribution function of slope −*a* can be obtained through data fitting, as shown in Figure 12b.

(c) The power-law distribution. Based on Equation (3b) and Table 4, we obtained the inexact F-N curve associated with the parameter intervals of −*a* and C under the 95% confidence level.

The detailed fitting parameters of F-N curves are listed under different distributions in Table 5. According to Figure 12a,b, the distribution of slope −*a* can be obtained. In detail, the distribution of −*a* under the normal distribution is presented as follows:

(5a) $$\frac{1}{0.0915\sqrt{2\pi }}exp\left(-\frac{{\left(-a+1.3424\right)}^{2}}{2\times 0.0915^{2}}\right),$$

and under the improved normal distribution, it is presented as follows:

(5b) 0.0264+0.71250.1182π2exp(−2(−a+1.3701)20.11822).

Moreover, the F-N curve under the power-law distribution is presented with 95% confidence levels, as follows:

(5c) lgF=[−1.378,−1.229]lgN+lg[0.9757, 1.057].

It is shown that the *R*^2^ (i.e., coefficient of determination) obtained under the distributions of normal, improved normal, and power-law were 0.6422, 0.89952, and 0.9936, respectively. Moreover, we also compared the root-mean square error (RMSE) values that were used to measure the deviation between the projected values from the fitting curves and the observation values. The RSME values were 1.0921 under the normal distribution, 0.02306 under the improved normal distribution, and 0.0197 under power-law distribution, respectively. It is indicated that the fitting curve based on the power-law function was the best in performance from the perspectives of both *R*^2^ and RMSE.

**Figure 13:**
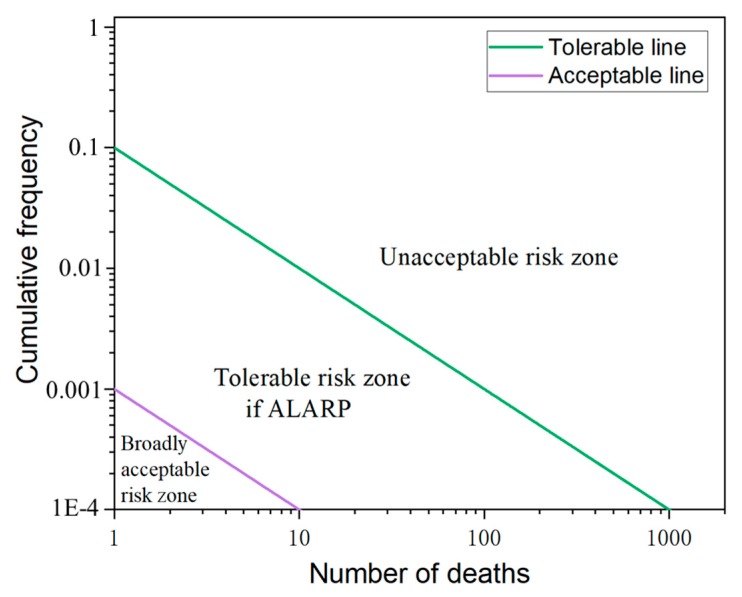
The risk criterion of the F-N curve in the logarithmic coordinate system. The area is divided into three zones by the tolerable and acceptable lines. ALARP principle is the abbreviation of as low as reasonably practicable principle.

The social risk of HTAs was further assessed using the proposed enhanced F-N curve under uncertainty, reflecting both the direct influence on casualties and the long-term harm to society. The social risk is usually evaluated based on the socially acceptable risk standard, such as the ALARP principle. For example, the ALARP principle divides the area that is formulated by the coordinate axes of casualty number and the associated occurrence frequency into the unacceptable risk, tolerable risk if ALARP, and broadly acceptable risk zones by the tolerable and acceptable lines [43]. Therefore, through assigning values to *a* and *C* in Equation (3a), the social risk of HTAs could be assessed with the utilization of the enhanced F-N curve. The tolerable and acceptable lines based on the F-N curve were obtained through deciding the slope of −*a* in Equation (3b), with *a* = 1 or 2 from existing studies [37]. Considering the influences of the HTAs, let *a* = 1 to reflect the neutral risk situation. The values of *C* in the equation represent the Y-intercept of the tolerable and acceptable lines, respectively. As for the tolerable line, *b* = 0.1 is taken in the study. Accordingly, the acceptable line was set along with the tolerable one according to the ALARP principle, and the Y-intercept was 1% of that from the tolerable line that was *b* = 0.001. The detailed unacceptable, tolerable if ALARP, and broadly acceptable risk zones associated with the tolerable and acceptable lines are displayed in Figure 13.

**Figure 14:**
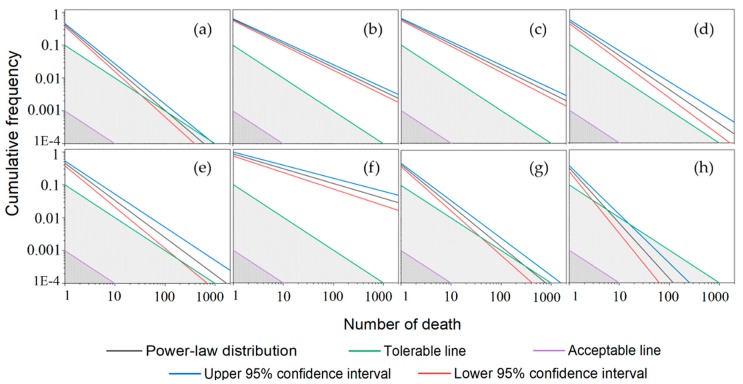
The F-N curves under uncertainty for different data sets, which were (**a**) 371 HTAs from 2004–2018 in China; (**b**) 1217 HTAs on road in 95 countries; (**c**) 715 HTAs on the railway in 95 countries; (**d**) HTAs in the counties of United States, Canada, Australia, Japan, New Zealand, and Norway; (**e**) HTAs in the European Union; (**f**) HTAs in the rest of the world with the exception of the United States, Canada, Australia, Japan, New Zealand, Norway, and the European Union; (**g**) the survey data of 322 hazmat accidents from road transport from 2000–2008 in China; and (**h**) 217 fatal HTAs from 2013–2017 in China.

The F-N curve under uncertainty could be developed under the power-law distribution for evaluating the severity of accidents based on the social risk criterion using the method proposed in the previous section. Besides the database used in the study, the other seven groups of data were digitized from references [8,9]. Among them, groups of (b) to (f) were from survey data of 1932 accidents (including 242 fatal accidents) that occurred during the land transport of hazardous material from 1931–2004 over 95 countries. Thus, a total of eight groups of data were used to help understand the applications of the proposed F-N curve method for severity analysis. The eight groups of data were as follows: (a) 371 HTAs from 2004–2018 in China; (b) 1217 HTAs on road in 95 countries; (c) 715 HTAs on the railway in 95 countries; (d) HTAs in the United States, Canada, Australia, Japan, New Zealand, and Norway; (e) HTAs in the European Union; (f) HTAs in the rest of the world with the exception of the United States, Canada, Australia, Japan, New Zealand, Norway, and the European Union; (g) survey data of 322 hazmat accidents from road transport from 2000–2008 in China; and (h) 217 fatal HTAs from 2013–2017 in China. The enhanced F-N curves under uncertainty associated with the lower and upper 95% confidence intervals, as well as the tolerable and acceptable lines, were obtained from the above data sets shown in Figure 14.

On a log–log basis of the power-law distribution, none of the F-N curves obtained fell into the acceptable risk zone that ranged below the acceptable line, as can be seen from Figure 14. It is indicated that the severity of the HTAs by road was relatively high for all the investigated cases. When a curve exists in the acceptable risk zone, it demonstrates that the corresponding risk may have an acceptable impact on the whole society, and there is no need to take extra measures to mitigate the risk generated by the HTAs. Furthermore, most of the curves appeared above the tolerable line, located in the unacceptable risk zone. It is indicated that the risks of HTAs had significant impacts on society, leading to immediate actions to control the related risks being needed. The risks of HTAs that occurred in China do not seem more severe than the other countries/regions based on the investigated data analysis with the layout positions. Specifically, the risk tended to be lower by the curve slope compared with Figure 14g,h.

In Figure 14, the black line represents the ordinary F-N curves, while the proposed method allows the local authorities to consider the risk uncertainty presented by the upper and lower 95% confidence intervals. The area between the tolerable and tolerance lines is the zone wherein the impact of risk is not unusually large or tolerable according to the aforementioned ALARP principle. However, it cannot be ignored, and is generally maintained at a level that is reasonably feasible with less social losses. The risk assessment of accidents can lead to different results considering the existing uncertainty. For example, as for Figure 14a, the risk of HTAs would be acceptable when the number of deaths exceeds 100 based on the ordinary F-N curve (i.e., the black line). The acceptable risk could be over 800 fatalities from a pessimistic attitude. On the contrary, according to an optimistic attitude, the risk of accidents with more than 40 deaths would be already small. Similarly, the risk could be acceptable when the fatalities are more than 40 for Figure 14g. As shown in Figure 14h, the optimistic risk assessment could be acceptable when fatalities exceed three. While making decisions with a pessimistic attitude, the risk would be relatively low when the number could exceed 18, based on the investigated data from 2013–2017 in China.

## 8. Discussion

The study highlights the need for the evaluation of accidents and continuous improvements in risk management for hazmat transportation. Thus, a comprehensive analysis framework for characteristic analysis, cause identification, and severity assessment was proposed and applied to a database of 15 years of fatal accidents of Levels II to V, which accounted for 14% of the total accidents caused by hazmat road transportation. Although the research mainly focused on the study case of China, the results suggest that the proposed methodology framework consisting of characteristics, causes, and severity analysis methods for the HTAs can offer the opportunity to be applied to many other safety management practices. For example, as for the HTA database of a specific country (or a region), the characteristics of HTAs can be analyzed first to obtain the necessary information, such as frequency, temporal and spatial trends, as well as a consequence. Then, the decision tree-based cause–consequence analysis method can be used to illustrate the complex relationship between causes and consequences. Through the severity analysis, decision-makers can be aware of HTA risk levels of the country (or region) by combining the ALARP principle with an enhanced F-N curve, while taking into account uncertainty. According to the severity assessment, if the social risk of HTAs is not acceptable, stricter policies for hazmat transportation management are desired to be implemented based on the characteristics and cause–consequence analysis. It can make policies more rational, as emphasis is placed on the management of critical aspects/factors that have more significant impacts on the consequences of HTAs. Otherwise, the current policies may not necessarily be adjusted.

Concerning the inherent uncertainty in severity analysis, the study proposed a method for establishing an enhanced F-N curve. Thus, the possible severity of hazmat accidents by road transportation could be evaluated under certain confidence levels, which has been ignored in previous research. When the F-N curve locates in the unacceptable risk zone, it means immediate and strict preventative measures are required, and the measures can be inspired and incorporated by the study findings from the characteristics and causes analysis of HTAs. In contrast to the conventional deterministic F-N curves, the study provides the empirically supported theoretical basis for severity analysis under various scenarios.

From the perspective of data analysis, as the application of machine learning techniques is desired and challenging in the field of HTA, multiple machine learning methods are introduced into the proposed characteristics–cause–severity analysis method framework for hazmat transportation. The *k*-means clustering approach was used for examining the spatial characteristics. The decision tree method was employed for revealing the complex relationships among the accident consequence and causes in terms of the key influencing factors of HTAs. For the method selection of the cause–consequence analysis, several methods proposed by literature were compared. In the study, the multinomial logit model was also tested for cause–consequence analysis purposes. Unfortunately, only the prediction accuracy of Level II was above 50%, and those of the other three levels were less than 5%. The significant result of the likelihood ratio was above 0.1, which indicates that the model cannot pass the test. The poor performance of the multinomial logit method could be attributed to the strong assumptions (i.e., the linear form of the utility function and the distribution assumption of errors), which make it unacceptable while applying to the HTA database. One of the aims of the study was to propose an accurate and non-biased method capable of HTA prediction. The Bayesian network was not suitable for the aim and the investigated data set due to a great deal of conditional probability estimation work and potential expert scoring. For the method assessment, the accuracy associated with the confusion matrix was introduced to measure the percentage of cases correctly classified by the classifier. In the investigation from existing studies, the prediction accuracy was acceptable when it exceeded 50% [28,29,44]. Thus, the decision tree model proposed in the study achieved a relatively satisfying performance in the cause–consequence analysis. 

There are still some limitations of the study in the following aspects. First, HTA-related studies require high-quality information, but the specialized databases are not yet available for the public; thus, more complete HTA data can provide a comprehensive understanding of the status of hazmat transportation management. Second, although the result of the decision tree-based cause–consequence analysis was acceptable, improvements on data information and modeling methods may increase the accuracy. Third, multiple sources of databases in various countries regarding HTAs are still required to help enhance the proposed method in real-world applications. Therefore, enriching HTA databases, proposing more advanced analysis techniques, and improving the existing methods are desired for better conducting the characteristics, cause, and severity analysis for hazmat transportation risk management in the future.

## 9. Conclusions

Due to the inherent physical and chemical instabilities of hazmats, accidents are more likely to occur during the process of road transportation. HTAs may cause catastrophic losses of lives and economics, as well as damages to the environment. The study proposed an accident characteristic–cause–severity analysis method for hazmat transportation management. A survey on 371 HTAs with consequence Levels II to V involving road transportation in China, which were collected from possible public and professorial sources between 2004–2018, were conducted. The study firstly presented a descriptive statistical analysis of the trends and the spatial–temporal characteristics of the fatal HTAs. It can be concluded: (1) hazmats of Class 3 associated with oil lead to the most HTAs among all the hazmat species, and collision and non-collision accidents account for 57.68% and 42.59% of accidents, respectively; (2) the highest accident rate of unit freight volume is in February; and (3) the regional population, GDP, road freight volume, and length of road network are relevant with number of HTAs, and there is a higher proportion (30.73%) in eastern China. The decision tree approach using CHAID operator was developed for the quantitative description of the relation among the hazmat properties, accident characteristics, and the consequences of HTAs. Subsequently, an enhanced F-N curve method based on power-law distribution was proposed for addressing the uncertainty and was applied to several practical data sets for severity analysis. Through the introduction of the ALARP principle for determining the acceptable and tolerable levels, these results presented by F-N curves were compared for understanding the severity and the associated implications. It is indicated that the F-N curves are above the tolerable line for most hazmat accident scenarios.

## Figures and Tables

**Table 1:** Results of Tukey’s test when *k* = 4 and 5 with the significance level of 0.05.

Cluster	Group			
	1	2	3	4
*k* = 4				
Cluster 1	−1.1561			
Cluster 2		−0.1744		
Cluster 3			0.9926	
Cluster 4				1.6818
P				
*k* = 5				
Cluster 1	−1.1561			
Cluster 2		−2.0040		
Cluster 3			0.6086	
Cluster 4			1.0502	
Cluster 5				1.9426
P			0.143	

Note: The values in the table are the average comprehensive factor scores of each cluster.

**Table 2:** The inputs of classification of independent variables for the decision tree.

Variable	Value	Number after Separated
**Time of accident**		
Early morning, from 12 am to 6 am	1	226
Forenoon, from 6 am to 12 pm	2	128
Afternoon, from 12 pm to 7 pm	3	145
Evening, from 7 pm to 12 am	4	70
**Class of hazmat**		
Explosive substance and articles	1	12
Gases	2	108
Flammable liquids	3	317
Flammable solids, self-reactive substances, substances liable to spontaneous combustion, and substances which in contact with water emit flammable gases	4	11
Oxidizing substances and organic peroxides	5	3
Toxic substances and infectious substances	6	34
Radioactive materials	7	0
Corrosive substances	8	84
Miscellaneous hazardous substances	9	0
**Type of accident**		
Collision	1	352
Non-collision	2	217
**Specific type of accident**		
Collision with fixed objects	1	18
Collision with non-motor vehicle	2	6
Collision with parked vehicle	3	12
Two-vehicle head-on	4	60
Two-vehicle sideswipe	5	16
Two-vehicle rear-end	6	179
Multivehicle collide	7	57
Rollover	8	135
Run-off-road	9	49
Tank and safety accessory failure	10	8
Vehicle-related defect	11	17
Others	12	12
**F** **actor of accident**		
Environment	1	50
Management failure	2	42
Equipment malfunction	3	44
Driver error	4	433
**Level of accident**		
Level II	1	408
Level III	2	104
Level IV	3	32
Level V	4	25
**Level of Road**		
Highway and other high-level roads	1	493
Urban road	2	37
Rural road	3	39

**Table 3:** The confusion matrix of the decision tree method.

Actual Value	Prediction				
	Level II	Level III	Level IV	Level V	Correct Prediction Percentage
Level II	399	2	0	7	97.8%
Level III	93	9	0	2	8.7%
Level IV	19	1	0	12	0.0%
Level V	4	1	0	20	80.0%
Overall percentage	90.5%	2.3%	0.0%	7.2%	75.2%

Notes: In the confusion matrix, each row of the matrix represents the instances in a predicted class, while each column represents the instances in an actual class (or vice versa). Dividing the number of correct predictions (the sum of the numbers on the diagonal) by the total number of inputs was equal to 75.2%.

**Table 4:** The data for the F-N curve under uncertainty.

$\mathrm{Number}\;\mathrm{of}\;\mathrm{Death}\;(N_{i})$	Number of HTAs	$\mathrm{Cumulative}\;\mathrm{Probability}\;(F_{j})$	$\mathrm{Lg}\;N_{i}$	$\mathrm{Lg}\;F_{j}$	$\mathrm{Slope}-a_{i}$
1	193	1.000	0	0	NA
2	91	0.480	0.301	−0.319	−1.060
3	28	0.235	0.477	−0.630	−1.320
4	21	0.159	0.602	−0.799	−1.326
5	8	0.102	0.699	−0.990	−1.416
6	4	0.078	0.778	−1.107	−1.423
7	5	0.067	0.845	−1.171	−1.386
8	3	0.054	0.903	−1.268	−1.404
9	3	0.046	0.954	−1.339	−1.403
11	1	0.038	1.041	−1.423	−1.367
12	4	0.035	1.079	−1.455	−1.349
15	1	0.024	1.176	−1.615	−1.373
17	1	0.022	1.230	−1.666	−1.354
20	1	0.019	1.301	−1.724	−1.325
29	2	0.016	1.462	−1.791	−1.225
36	1	0.011	1.556	−1.967	−1.264
40	1	0.008	1.602	−2.092	−1.306
41	1	0.005	1.613	−2.268	−1.406
58	1	0.003	1.763	−2.569	−1.457

Notes: F-N curve presents the relationship between the cumulative probability *F* and the number of deaths *N*, and slope −*a* is equal to lg *F* divided by lg *N*.

**Table 5:** Parameter estimations under different distributions.

Distribution	Normal Distribution	Improved Normal Distribution	Power-law Distribution
Parameters and 95% confidence interval	*μ* = −1.3424 σ = 0.0915 (−1.5217, −1.1631) for *μ*	*μ* = −1.3701 σ = 0.1182 (−1.3772, −1.3630) for *μ*	−a = (−1.378, −1.229) C = (0.9757, 1.057)
*R* ^2^	0.6422	0.8995	0.9936
RMSE	1.0921	0.0231	0.0197

Note: *R*^2^ shows the degree of fitting of the regression line to the observed values. RMSE is the abbreviation of root-mean square error, which can reflect the fitting accuracy.
